# The Works of Matthew Baillie, M.D. with an Account of His Life, Collected from Authentic Sources

**Published:** 1826-01

**Authors:** 


*The Works of Matthew Baillie, M.D. to which is prefixed an Account of his Life, collected from authentic Sources.* By JAMES WARDROP, Surgeon Extraordinary to the King, &c. &c. London. 2 vols. 8vo. Longman & Co. Paternoster Row.

THE first of these volumes contains the life and miscellaneous papers of the late Dr. Baillie; the former occupying 60, and the latter 237 pages. The second volume contains the "Morbid Anatomy of some of the most important Parts of the Human Body," which extends to 407 pages, and is preceded by a dissertation on diseased structures, by Mr. Wardrop, occupying 39 pages. In an advertisement prefixed to the first volume, Mr. Wardrop informs us that his object in undertaking the duty of editor on this occasion, has been to collect together all the works of Dr. Baillie, consisting of some which had appeared in various publications during the author's life, and of a few which were found among his MSS. after his decease.

The reader, who is not already acquainted with the history of Dr. Baillie, will learn from Mr. Wardrop's very interesting account, by what a fortunate concurrence of circumstances he was raised to the singular eminence which he attained. Adopted as a son, and educated under the immediate care of his uncle, Dr. William Hunter, one of the most distinguished of British physicians, Dr. Baillie entered upon his professional career with many unusual advantages. His mind, however, had been too well disciplined in early life, under the able superintendance and anxious care of his father, (a clergyman of the church of Scotland, and an eminent professor in the University of Glasgow,) and he possessed too much original modesty and sobriety of character, to be disposed to rely upon such extraordinary advantages, to the exclusion of personal exertions, as young persons differently constituted might have been led to do. On the contrary, while he judiciously availed himself of the favourable circumstances which fortune had placed within his reach, he never lost sight for a moment of the value of his own exertions, but prudently laboured to perfect his professional acquirements with as much, nay, perhaps with more assiduity, than if he had had nothing save his personal qualifications to confide in for his advancement in the world. The remarkable success which he met with affords a striking illustration of the truth of the doctrine which considers labour judiciously bestowed, as the source of wealth. There is so much of truth and good sense in the following observations of Mr. Wardrop on this subject, that we cannot refrain from giving them a place here.

"In no department of life do men rise to eminence who have not gone through a severe course of study, and elaborate preparation, for, whatever may be the difference in the original capacities of individuals, it is the cultivation of the mind alone which elevates to distinction. No man laboured more in early life than Dr. Baillie, in order to acquire what may be said to have been the ground-work of his professional fame; and his mind thus received that general tuition which fitted it in an especial manner successfully to prosecute the study of medicine. Men sow the seeds of their future reputation, perhaps, at a much earlier period than is usually supposed, and the latter years of life are occupied merely in digesting and arranging what was in early years impressed. It is, therefore, an erroneous doctrine that the student of medicine should trust to experience for the acquirement of useful knowledge. Experience is too apt to be confounded with observation, and, in contemplating the life of Dr. Baillie, it is evident that all he did for medical science was accomplished before he had reached his fortieth year, and before he could have had that experience which is generally supposed necessary to lead to eminence."

Mr. Wardrop likewise remarks that there is no profession in which the progress even of the best qualified is so slow as that of medicine, because success depends so much upon the exertion and assiduity of the individual; and it is a curious fact, that, notwithstanding the very auspicious circumstances which favoured Dr. Baillie, he was nearly forty years of age before he found himself fully established in practice.

The following passage, containing the sentiments of a man so eminent as Dr. Baillie, on the difficulties which beset medical men in their progress in the world, cannot fail to be read with interest.

" He used to narrate, in the most open manner, the history of his own life, and to describe to the younger members of the profession, the rocks and shoals which he had met with, contrasting these with his long looked for, but ultimate success. Scarcely any medical person commenced practice in London without being introduced to him; and such introductions usually led him to make some observations on what his own experience had shown him to be the necessary qualifications to ensure their success, and the probable progress they must expect to make in their professional career. He pointed out the necessity of competency, of integrity, and of industry, and the slow progress of the most eminent men who had gone before them; and, on the other hand, the transitory fame of all those who had ever attempted to gain professional reputation as if by storm. Such observations, coming from such an authority, were of the greatest use in checking the too warm imagination of the youth, and thus enabled him to see his situation in life, as it really was, and not, perhaps, as he had allowed his imagination to paint it. Again and again I have heard him remark that he never knew an instance of a practitioner settling in London, who made a large income at first, continuing afterwards to do so. I have been informed by one of the intimate companions of his early days, that he long considered his own success as hopeless, and never contemplated acquiring any thing like celebrity, or even competency. He used, in pointing out the difficulties in the road to medical fame, which he had often occasion to do, to impress on young men the impropriety of living expensively, and the error of considering equipages, and parade, and entertainments necessary for success, candidly illustrating these opinions by his own experience.

As the biographer of Dr. Baillie, Mr. Wardrop has acquitted himself in a manner at once highly creditable to his talents as an author, and remarkably characteristic of his own goodness of heart. He has properly placed in a conspicuous point of view those traits in the character of that excellent person to which he was chiefly indebted for the great eminence he deservedly attained, as the first medical authority in this country; but we are better pleased with the zeal and ability with which Mr. Wardrop seizes every opportunity of impressing upon his readers how much Dr. Baillie owed his great reputation and success to his moral worth and inflexible integrity, to the laudable anxiety which he constantly evinced to protect the characters and interests of others, and to the unconstrained simplicity and unassuming urbanity of his manners towards his professional brethren. As described by Mr. Wardrop, the life of Dr. Baillie is a moral lesson, calculated to produce the best effects, holding up to the younger members of the profession those social virtues, the exercise of which give a zest to the enjoyment of human life, and moral rectitude of principles, as qualities more worthy of their emulation than even the highest professional acquirements.

We do not mean at present to notice any of Dr. Baillie's miscellaneous papers, which have long been in print, though scattered through various publications. Mr. Wardrop has certainly rendered a service to his readers by collecting them into one volume. They occupy 189 pages, and the remainder of the first volume, 45 pages, is filled with accounts of dissections now printed for the first time. The chief merit of them appears, to us, to consist in the conciseness and clearness with which the most remarkable facts are noted, rendering them worthy, in this respect, of serving as models for recording facts and observations of the same description.

One of these dissections shews that the rete mucosum of the negro, notwithstanding what has often been said to the contrary, is sometimes regenerated. Another, is an instance of an ulcer through the coats of the stomach, situated near the small curvature, and large enough to have easily admitted the end of an adult fore-finger. The lips surrounding it were not much ragged, so that it appeared to have been of some standing. Food was not found in the abdomen, because it was taken in very small quantity, and naturally fell to the great end of the stomach as the most depending part. There is a case of a septum dividing the urinary bladder into two chambers: Dr. Baillie observes that, in such a case, one chamber might be emptied by a catheter and not the other, or that, the one being emptied, the symptoms of retention might still remain. In one instance, Dr. Baillie states that, besides other marks of disease of the arterial system, he found the left carotid artery obliterated, and it is curious to see how this eminent man reasoned from this then singular fact, as to the probable results of an operation unheard of at that time, but which has since been repeatedly performed with success, and which, with the application of ligatures to other large arteries, may justly be regarded as one of the grandest improvements in the art of surgery, for which the world is indebted to the zeal and energy of British surgeons.

"This case shews," says Dr. Baillie, "that an animal may live without circulation through one carotid artery, so that it may be tied or obliterated without life being certainly endangered. It also shews how an aneurism may cure itself, for if an aneurismal dilatation be entirely plugged up, so that there is no circulation through it, there is no cause existing to increase the dilatation, and, therefore, it will remain stationary, or be diminished by absorption."

A case is given of a woman who had died suddenly undelivered, and whose body was opened half an hour after death, in order to recover the child. The means used for that purpose failed, but Dr. Baillie mentions that the uterus contracted to one half its natural size after it was taken out of the body; a proof, as he remarks, of its muscularity.

The following case, of "bone formed in different organs," being very singular, we here insert at length.

"A patient was admitted into St. George's Hospital with a hard swelling of his knee, for which the leg was amputated. The stump was going on well for some time, and his general health seemed very good, when he was suddenly seized with difficulty of respiration, and at length he died. On opening the body, great irregular knobs of bone were found in the substance of the lungs, and, on the back part, a knob of bone fixed to the ribs. Bony matter was found to be secreted on the surface of the sore, when the knee of the amputated leg was examined; the tumour, which was as large as the head of an adult, was found to be a bony tumour, and it had only been about twenty weeks in growing to that enormous size. There had been a very strong disposition in the constitution to form bone. The system had indulged this disposition in the knee, when it was disappointed in this by amputation; it transferred the process to the lungs, a vital part, by which the man lost his life."

We have already stated that the "Morbid Anatomy" occupies the principal part of the second volume. This valuable work established Dr. Baillie's reputation during his life, and on it undoubtedly must rest his posthumous fame. Its form and execution are both strikingly characteristic of the modesty and good sense for which the learned author was so remarkable. In composing that work, his aim seems to have been to communicate all that he intended in the fewest words and least expensive form. He accordingly limits himself to a succinct description of those morbid structures which had actually been observed, and of the most remarkable and uniform symptoms usually accompanying them. Had he professed to compose a general system of the morbid anatomy of the human body, his task would have been much more difficult, he must have made arrangements founded upon principles the propriety of which would have been liable to be questioned, and repetitions could not well have been avoided; but he could not have communicated more knowledge than the work contains in its present form, while, from the imperfect information then and even now possessed upon many parts of the subject, he must of necessity have left his work incomplete as a system. His style is perspicuous and concise. Like Lord Bacon and Sir Isaac Newton, the best of philosophical writers, as well as the greatest of philosophers, he rejects, as much as possible, all technical phraseology and scholastic terms.

The great estimation in which this excellent work was deservedly held, may be judged of by the fact of its having been speedily translated into the French, Italian, and German languages. The illustrious Professor Soemmering, who conferred a favour on his countrymen and did honour to himself, as well as to Dr. Baillie, by translating it into the German, speaks of its merits in the following terms.

"The strictest attachment to truth characterises every page of Dr. Baillie's work, accurate and impartial reasoning is every where conspicuous, and there is no part but what displays the share of attention that had been paid in observing those alterations of structure to which the various parts of the body are subject. Attentive and thinking practitioners will here find facts which will furnish them with the true causes of many phenomena they have observed; they will often find explanations they had long wished for, and some will meet with facts which, instead of agreeing with favourite theories, will serve, in the strongest manner, to refute them."

"No one," observes Mr. Wardrop, "can form a just estimate of his (Dr. B's.) power of compressing matter, without comparing his Morbid Anatomy with the voluminous works on the same subject which preceded it; or by attempting to add to it from the writings both previous to and since its publication. This is strikingly illustrated by the little importance that can be attached to the notes of the different translations of that work, and there is scarcely a pathological fact in the stupendous folios of Bonetus, Lieutaud and Morgagni, which is not to be found in Dr. Baillie's small volume."

Mr. Wardrop was probably led by experience to make this remark, as he has had occasion to add several articles to this edition of the Morbid Anatomy. These new articles are interspersed through the work, being introduced where the other diseases of the several parts to which they relate are treated of. They consist of the following :—Accumulation of air in the cavity of the pericardium—unusual fatness of the heart—concretions in the cavities of the heart—excrescences on the valves of the heart—indurations of the parietes of the heart—flaccidity of the parietes of the heart—concretions in the thorax—granular tubercles of the lungs—melanoid tubercles of the lungs—warts on the larynx—diseases of the omentum—a cul-de-sac in the intestines—extraneous bodies, causing death, in the appendix vermiformis—fungus hæmatodes of the mesenteric glands—schirrous tubercles of the liver—hæmatoid tubercles of the liver—melanoid tubercles of the liver—fungus hæmatodes of the bladder.

Dr. Baillie's detailed description of the diseased appearances of the different organs is preceded, as we have already mentioned, by an introductory dissertation by Mr. Wardrop, entitled "Preliminary Observations on Diseased Structures." Mr. Wardrop was induced to think that these observations might prove useful, as this branch of medical science has been cultivated with great zeal on the Continent, as well as in this country, since the publication of Dr. Baillie's work; and being manifestly the fruit of much reflection on the part of Mr. Wardrop, we trust they may prove useful, by inducing others also to reflect on the important subjects treated of.—We think, however, that the able writer might with more propriety have entitled them General Observations on Diseases. He tells us, for instance, that "some diseases go through a particular course, and leave the body uninjured." Surely this has nothing to do with diseased structures, yet in the next paragraph he says, "I ought to observe that what has now been said is meant to apply only to diseased structures, the department of medical science, on which alone Dr. Baillie has professed to treat," and then proceeds to speak of "other phenomena attending particular diseases not coming within the province of pathological anatomy."

Mr. Wardrop's style of composition is generally correct and concise without being obscure, which, with simplicity, constitute, in our opinion, the characteristics most suitable for writings on medical subjects. We would prefer, however, some circumlocution to a want of perspicuity, the most essential ingredient of good style, and were not a little surprised to meet with the following incorrect sentence in Mr. Wardrop's observations on specific diseases. After stating that schirrus, fungus hæmatodes, fungus melanodes, scrofula, gout, scurvy, small-pox, measles, scarlatina and syphilis, are examples of specific diseases, he says, "diseases arising from a morbid poison are also specific; such as hydrophobia, and the various animal, vegetable, and mineral poisons, each of which produce (produces) specific effects on the human body." Independently of the grammatical inaccuracy which we have marked, we have here "the various animal, vegetable, and mineral poisons," classed with hydrophobia, as if they were diseases proceeding from morbid poisons, while, in fact, neither the vegetable nor mineral poisons could with propriety even be termed "morbid poisons." We were likewise surprised to find bubo classed by Mr. Wardrop among the secondary symptoms of syphilis. Were it possible to doubt, in cases where chancres have existed, that bubo is the effect of the irritation excited locally in the lymphatic gland, or to suppose that it is a symptom or consequence of constitutional contamination, what shall be said to those cases, of which we have met with one in our own practice, where venereal bubo occurs as the first symptom of the disease, and without having been preceded by chancre at all? In the case to which we allude, the subsequent appearance of venereal blotches, sore throat, and other distinctly marked secondary symptoms, left no room to doubt the nature of the disease.—In this arrangement of bubo among secondary symptoms Mr. Wardrop differs from Hunter, Howard, Swediaur, and indeed from every systematic author with whose opinions we are acquainted.

Mr. Wardrop treats of melanoid tumours and tubercles, which have heretofore been generally regarded as varieties of the fungus hæmatodes, as a distinct disease, under the term fungus melanodes, and, as this arrangement was new to us, so we presume it will be to some of our readers. We shall therefore submit to them Mr. Wardrop's observations, with only one remark, that the distinction appears not to have been made without due consideration of all the facts which are known to the profession on the subject.

"The structure of the fungus melanodes in many respects resembles that of fungus hæmatodes, the more striking difference consisting in the dark brown or black colour of the one, contrasted with the pale white or ash colour of the other.

"On dividing a melanoid tumour, its section presents, like the hæmatoid, a smooth unctuous surface, a quantity of the soft matter which enters into its composition soiling the knife. Sometimes the whole surface presents a uniform dark-coloured homogeneous mass, and sometimes it consists of a greater or less proportion of the dark matter, mixed in patches or in streaks with a pale-coloured medullary substance.

"The colouring matter can be separated by maceration from the rest of the diseased mass; and, when it is washed away, a structure more or less firm remains, distinctly circumscribed, and insulated from the adjacent healthy structure. The black matter mixes readily with water, and stains paper or the hand precisely the colour of Indian ink. It has no smell, and seems more to resemble a secretion than a decomposition; it is, indeed, in every respect, like the black pigment of the choroid coat, as the colouring matter secreted in large quantities by the scuttle-fish. By an accurate examination of this colouring matter and the black pigment through a microscope, they appeared to me quite analogous.

"When chemically examined, Breschet states, that the melanoid tumour very much resembled blood in its composition; consisting, 1st, of a coloured fibrine—2nd, of a black colouring matter, soluble in diluted sulphuric acid, and in a solution of the subcarbonate of soda; rendering these liquids of a red colour—3rd, of a small quantity of albumen—4th, of subcarbonate of soda, phosphate of lime, and oxyde of iron.

"The fungus melanodes undergoes a process of disorganization similar to the fungus hæmatodes, portions becoming softer, and the integuments covering it giving way; from such ulcerated openings there grows out a dark-coloured fungus. Whilst the primary tumour increases, sooner or later, the secondary symptoms of the disease make their appearance in like manner, as has been observed of other specific diseases, and these symptoms consist either in the glands, in the immediate vicinity of the primary tumour, becoming contaminated, or in melanoid tubercles forming in remote organs.

"Fungus melanodes has been observed in most of those organs liable to be affected with fungus hæmatodes. The skin, the thyroid gland, the uterus, the ovaria, the mamma, the eye-ball, have each been found affected with fungus melanodes in its primary form; whilst the lungs, the liver, the kidneys, the omentum and mesentery, the brain and the heart, the medullary membrane of the bones, and the cellular membrane, have been the seat of melanoid tubercles, or of the secondary symptoms.

"The melanoid tumour is by no means rare, and specimens of it are to be found in almost every museum. Dr. Baillie, however, does not seem to have ever remarked it. It has also been observed in several domestic animals, particularly in the horse.

"The fungus melanodes chiefly occurs in those advanced in life; whereas I have already stated, that fungus hæmatodes, like scrofula, was to be considered as a disease of infancy and youth. In this respect, therefore, the melanodes has a greater analogy to cancer than to hæmatodes. Like both cancer and fungus hæmatodes, it is accompanied by constitutional symptoms, and often destroys life without creating much manifest disturbance."

In conclusion, we have only to say that Mr. Wardrop has conferred an obligation on the profession, by the great pains he has evidently taken to render complete this edition of the "Morbid Anatomy of the Human Body," and that the little that Dr. Baillie has left it possible to add, by way of improvement, has been done in a manner altogether worthy of the original work, and well calculated to sustain Mr. Wardrop's reputation as an able and correct pathologist, which he established in early life by his justly esteemed Essays on the Morbid Anatomy of the Eye, as well as by his Treatise on Fungus Hæmatodes.

